# Differences in pandemic influenza vaccination policies for pregnant women in Europe

**DOI:** 10.1186/1471-2458-11-819

**Published:** 2011-10-20

**Authors:** Johannes M Luteijn, Helen Dolk, Gordon J Marnoch

**Affiliations:** 1 EUROCAT Central Registry, Institute of Nursing Research/School of Nursing, University of Ulster, Jordanstown Campus, Shore Road, Newtownabbey, BT37 0QB, UK; 2 School of Criminology, Politics and Social Policy, University of Ulster, Jordanstown Campus, Shore Road, Newtownabbey, BT37 0QB, UK

## Abstract

**Background:**

An important component of the policy to deal with the H1N1 pandemic in 2009 was to develop and implement vaccination. Since pregnant women were found to be at particular risk of severe morbidity and mortality, the World Health Organization and the European Centers for Disease Control advised vaccinating pregnant women, regardless of trimester of pregnancy. This study reports a survey of vaccination policies for pregnant women in European countries.

**Methods:**

Questionnaires were sent to European competent authorities of 27 countries via the European Medicines Agency and to leaders of registries of European Surveillance of Congenital Anomalies in 21 countries.

**Results:**

Replies were received for 24 out of 32 European countries of which 20 had an official pandemic vaccination policy. These 20 countries all had a policy targeting pregnant women. For two of the four countries without official pandemic vaccination policies, some vaccination of pregnant women took place. In 12 out of 20 countries the policy was to vaccinate only second and third trimester pregnant women and in 8 out of 20 countries the policy was to vaccinate pregnant women regardless of trimester of pregnancy. Seven different vaccines were used for pregnant women, of which four contained adjuvants. Few countries had mechanisms to monitor the number of vaccinations given specifically to pregnant women over time. Vaccination uptake varied.

**Conclusions:**

Differences in pandemic vaccination policy and practice might relate to variation in perception of vaccine efficacy and safety, operational issues related to vaccine manufacturing and procurement, and vaccination campaign systems. Increased monitoring of pandemic influenza vaccine coverage of pregnant women is recommended to enable evaluation of the vaccine safety in pregnancy and pandemic vaccination campaign effectiveness.

## Background

On 24 March 2009, an outbreak of novel H1N1 influenza A, now commonly referred to as the 2009 H1N1 pandemic influenza or simply the swine flu, was identified in Mexico. The new virus quickly spread and on the 11^th ^June 2009, the World Health Organization (WHO) raised the pandemic level to 6, indicating the first widespread influenza pandemic since the 1968 H3N2 Hong Kong flu. The European Centers for Disease Prevention and Control (ECDC) and the WHO defined clinical and pharmacological advisory management guidelines for managing this pandemic, while European national authorities were responsible for developing national vaccination policies.

During seasonal influenza and previous influenza pandemics, pregnant women have been at increased risk of adverse health outcomes [1,2]. Preliminary data collected between April 2009 and July 2009 from Australia and the USA suggested pregnant women in the second or third trimester who had H1N1 influenza, especially if suffering from co-morbidities, were particularly vulnerable to mortality and severe morbidity [3,4]. Both the European Commission (EC)[5] and the WHO [6] advised vaccination of pregnant women against the 2009 H1N1 pandemic swine flu, regardless of stage of pregnancy.

While policy advice was based on the available safety information in 2009, pharmacovigilance studies will need to verify the presumed safety of the pandemic vaccine and the adjuvants used. European Surveillance of Congenital Anomalies (EUROCAT), a network of population based congenital anomaly registers in Europe currently surveying 31% of European Union births [7], is participating in safety monitoring of the pandemic vaccines use in pregnancy with regard to congenital anomalies. This study reports the results of a survey undertaken to gather data regarding when and where pregnant women, and women of childbearing age (WCBA), received pandemic influenza vaccine, in order to interpret EUROCAT prevalence data on congenital anomalies. WCBA are relevant for this study since women may receive the vaccine before they know they are pregnant. The purpose of this paper is to describe the vaccination policies in European countries to enable discussion and reflection on the differences between pandemic vaccination policies and vaccination coverage of pregnant women between European countries.

## Methods

A questionnaire regarding vaccination policy and antiviral use was sent out to EUROCAT registry leaders April 23^rd^, 2010, with a reminder on May 18^th^, 2010. EUROCAT registries are situated in 17 EU countries and in Croatia, Norway, Switzerland and Ukraine. The EUROCAT-questionnaire contained eight questions with regard to antiviral use and vaccination policy and offer/uptake for pregnant women and WCBA. The majority of EUROCAT registry leaders who completed the questionnaire were public health specialists and clinicians who did not have specific responsibilities related to vaccination policy in their countries. Respondents were asked to estimate vaccination coverage of pregnant women in their areas using broad bands (0-24%, 25-49%, ...) with free space for more specific information and references where further data could be obtained. Some EUROCAT registries also collect individual exposure data on congenital anomaly cases but this was not yet available at the time of the survey.

Within the European regulatory system it is possible to collect or exchange information from other member states by circulating a request for Non-Urgent Information (NUI) between the competent authorities and the European Medicines Agency (EMA) [8]. The European regulatory system consists of the 27 EU member states plus Iceland, Liechtenstein and Norway. On July 23^rd^, 2010, a NUI was circulated to 27 Member States' representatives at the Pharmacovigilance Working Party (PhVWP) of the Committee for Medicinal Products for Human use (CHMP) of the EMA to collect information on pandemic vaccination policies and policies for antiviral use with regard to pregnant women and WCBA, to complement the EUROCAT survey. The NUI contained seven questions. The NUI requested weekly or monthly data on numbers of vaccinated pregnant women instead of banded coverage estimates. A second NUI was circulated on March 14^th ^2011 to confirm the data. In addition to the two survey questionnaires, we followed up any references given by respondents and conducted literature and conference abstract searches - eight additional relevant items were added from these sources [9-16].

### Role of the funding source

The study funder took no part in study design; in the collection, analysis and interpretation of data in the writing of the report; and in the decision to submit the paper for publication.

### Ethics

Under University of Ulster's ethical review policy this type of study does not require ethical review, and we have overarching ethical approval for activities carried out under EUROCAT.

## Results

A total of 16 countries of the 27 who were sent a NUI by EMA returned a complete response. One additional country sent a partial reply during the confirmation process. The completed EUROCAT questionnaire was returned from 17 of 21 countries covered by EUROCAT, giving a combined total of 24 responding countries (table 1). Of the 27 EU states Bulgaria, Cyprus, Estonia, Lithuania, Luxembourg and Romania were not covered by the combined replies.

**Table 1:** Response to the questionnaires by competent authorities and EUROCAT members

Country	Sources	
	**EUROCAT**	**EMA-NUI**

Austria	**⊠**	**□**

Belgium	**⊠**	**⊠**^1^

Bulgaria		**□**

Cyprus		**□**

Czech Republic	**⊠**	**□**

Croatia	**⊠**	

Denmark	**⊠**	**⊠**

Estonia		**□**

Finland	**⊠**	**⊠**

France	**⊠**	**⊠**

Germany	**⊠**	**□**

Greece		**⊠**

Hungary	**□**	**⊠**

Iceland		

Ireland	**⊠**	**⊠**

Italy	**□**	**⊠**

Latvia		**⊠**

Lithuania		**□**

Luxembourg		**□**

Malta	**⊠**	

Netherlands	**⊠**	**⊠**

Norway	**⊠**	**⊠**

Poland	**⊠**	**□**

Portugal	**⊠**	**⊠**

Romania		**□**

Slovakia		**⊠**

Slovenia		**⊠**

Spain	**⊠**	**⊠**

Sweden	**□**	**⊠**

Switzerland	**⊠**	

Ukraine	**□**	

United Kingdom	**⊠**	**⊠**

^1^Belgium replied to the second NUI circulated March 14^th^, 2011.

### Pandemic vaccination policies in Europe for pregnant women

Of the 24 European countries covered by the combined questionnaires, 20 countries (83%) reported having an official vaccination policy against pandemic swine flu for pregnant women (table 2). In 12 of these 20 countries, this policy did not include first trimester pregnant women (table 2). In Croatia, despite lack of an official policy, pregnant women in the second or third trimester of pregnancy could apply for vaccination via health insurance. In Czech Republic while there was no official vaccination policy, a small number of pregnant women were vaccinated-In Latvia and Poland there was no recorded vaccination of pregnant women.

**Table 2:** Vaccination policy overview by country for pregnant women and the general population

Country	Vaccination strategy for Pregnant women	Vaccination strategy for the general population
	Policy Trimesters Vaccines^1^	

	1 2 3	

Austria	Yes **⊠ ⊠ **Cev	The entire population was offered the vaccine and certain groups^4 ^were prioritized

Belgium	Yes **⊠ ⊠ **Pad	Priority groups only^4^

Czech Republic^2^	No	No

Croatia^3^	No	No

Denmark	Yes **⊠ ⊠ **Pad	Priority groups only^4^

Finland	Yes **⊠ ⊠ ⊠ **Pad	The entire population was offered the vaccine and certain groups^4 ^were prioritized

France	Yes **⊠ ⊠ **Pan	The entire population was offered the vaccine and certain groups^4 ^were prioritized

Germany	Yes **⊠ ⊠ **Pad, PIV [14]	The entire population was offered the vaccine and certain groups^4 ^were prioritized

Greece	Yes **⊠ ⊠ **Foc, Pad	The entire population was offered the vaccine and certain groups^4 ^were prioritized

Hungary	Yes **⊠ ⊠ ⊠ **Flu	The entire population was offered the vaccine and certain groups^4 ^were prioritized

Ireland	Yes **⊠ ⊠ **Cev, Pad	The entire population was offered the vaccine and certain groups^4 ^were prioritized

Italy	Yes **⊠ ⊠ **Foc	The entire population was offered the vaccine and certain groups^4 ^were prioritized

Latvia	No	No

Malta	Yes **⊠ ⊠ ⊠ **Pad	The entire population was offered the vaccine and certain groups^4 ^were prioritized

Netherlands	Yes **⊠ ⊠ **Foc	Priority groups only^4^

Norway	Yes **⊠ ⊠ **Cev, Pad	The entire population was offered the vaccine and certain groups^4 ^were prioritized

Poland	No	No

Portugal	Yes **⊠ ⊠ **Pad	Priority groups only^4^

Slovakia	Yes **⊠ ⊠ ⊠ **Pan	The entire population was offered the vaccine and certain groups^4 ^were prioritized

Slovenia	Yes **⊠ ⊠ ⊠ **Cev, Pad	The entire population was offered the vaccine and certain groups^4 ^were prioritized

Spain	Yes **⊠ ⊠ ⊠ **Pan	Priority groups only^4^

Sweden	Yes **⊠ ⊠ ⊠ **Pad	The entire population was offered the vaccine and certain groups^4 ^were prioritized

Switzerland	Yes **⊠ ⊠ **Cel, Foc	The entire population was offered the vaccine and certain groups^4 ^were prioritized

United Kingdom	Yes **⊠ ⊠ ⊠ **Cev, Pad	Priority groups only^4^

^1^Cel = Celtura^® ^(Novartis), Cev = Celvapan^® ^(Baxter), Flu = Fluval P^® ^(Omnivest), Foc = Focetria^® ^(Novartis), Pad = Pandemrix^® ^(Glaxo Smith Kline), Pan = Panenza^® ^(Sanofi Pasteur), PIV = CSL Pandemic Influenza Vaccine^® ^(CSL Biotherapies). Of these vaccines Celtura, Fluval P, Focetria and Pandemrix are adjuvanted while Celvapan, Panenza and CSL Pandemic Influenza Vaccine are non-adjuvanted. Celtura, Fluval P, Focetria, Pandemrix, Panenza and CSL Pandemic Influenza Vaccine contain Thiomersal while Celvapan does not contain Thiomersal.

^2^In Czech Republic the Pandemrix vaccine was unofficially recommended to pregnant women of 2^nd ^and 3^rd ^trimester and a small number of pregnant women were vaccinated. The vaccine was offered to the general population. There was no official vaccination policy.

^3^In Croatia, pregnant woman of 2^nd ^and 3^rd ^trimester and the general population could apply for vaccination with the Focetria vaccine via health insurance and a small number of pregnant women were vaccinated. There was no official vaccination policy.

^4^Priority groups include one or more of the following groups: pregnant women, health care workers, persons suffering from specific co-morbidities and/or people working in essential public services.

In total 7 different vaccines were used for pregnant women, of which 4 were adjuvanted and 3 were non-adjuvanted (table 2). Vaccination campaigns of pregnant women were initiated between 28^th ^September 2009 (Hungary) and 27^th ^December 2009 (Malta). (Data not shown) Respondents from eight countries were unable to provide detailed information on when the vaccination campaign of pregnant women started. Note the initiation of the vaccination campaign does not necessarily correspond to initiation of vaccination.

### Pandemic vaccination policies in Europe for women of childbearing age

WCBA were not distinguished from the general population in any country regarding pandemic vaccination policy. In 14 out of 24 European countries (58%) the pandemic vaccine was offered to priority groups first (table 2) and then offered to the general population. In six out of 24 European countries (25%) the pandemic vaccine was offered to priority groups only and not offered to the general population. In Croatia and Czech Republic, the general population could apply for vaccination despite the absence of an official vaccination policy. In Latvia and Poland, no vaccination took place.

### Data on exposure of pregnant women to swine flu vaccine

For five out of 20 countries with an official vaccination policy, no exposure data was available regarding pregnant women. Other countries had a variety of data including weekly or monthly vaccination rates or total number of pregnant women vaccinated or vaccine doses distributed, registers for pregnant women who had been given vaccine, surveys of general practitioners or monitoring of specific areas or hospitals (table 3). Vaccination uptake rates above 50% have been reported from 3 countries (Finland, Ireland and Norway; Table 3).

**Table 3:** Pandemic 2009 H1N1 influenza vaccination coverage of pregnant women; available data per European country

Country (births 2009*[34])	EMA-NUI	Information provided via EUROCAT	Other information
Austria (76033)		0 to 24%. Based on personal estimation	

Belgium (126886)	No exposure data available	Unknown to registry.	

Czech Republic (118283)	No exposure data available	Unknown to registry.	

Croatia (44794)		Only 20 pregnant women have undergone vaccination. Data from Croatian Institute of Public Health, Epidemiology unit	

Denmark (62831)	Weekly for Pandemrix: 5770 up to February 2010. WCBA: 42546 up to February 2010.	Unknown to registry.	

Finland (60187)	No exposure data available	51.2% of mothers giving birth October-December 2009 were vaccinated with Pandemrix according to the Finnish medical birth register.	

France (823925)	No exposure data available. WBCA: 980000 women aged 18-44 years old were vaccinated.	1) 25 to 49%. Based on CoFluPreg study (maternity wards in Paris) [12]. 2) Vaccination was not yet available at the time of pandemic wave (weeks 30-38) for Isle de la Reunion [35].	22.7% up to January 2010. Data from French State Health Insurance Fund [9]. 37.1% between Oct 2009 and Feb 2010. Data from COFLUPREG study [12].

Germany (664219)		0 to 24%. Based on registry and the Robert Koch Institute	8.8% between week 47/2009 and 14/2010. Based on 65 pregnant women [15].

Greece (118234)	Weekly for Focetria (1382 up to January 2010) and Pandemrix (751 up to January 2010)		

Hungary (96297)	17200 vaccinated		About 16% [10].

Ireland (73870)	Several cut-off points for combined Celvapan and Pandemrix vaccination doses (31093 by June 2010).	A cross-sectional telephone survey found an uptake of 67% in pregnant women (n = 1725)[36] while the National Summary of Pandemic Influenza Vaccination estimates a vaccination rate of 32.5% [37].	

Italy (570428)	Weekly for Focetria (23016 up to week 23/2010)		

Latvia (21708)	No vaccinations took place	No vaccinations took place	

Malta (4136)		0 to 24%. Based on national vaccination register data (numerator: persons classified as pregnant when vaccinated, denominator: number of persons pregnant on 1/1/2009 [data for 2010 not yet available]	

Netherlands (184641)	Focetria exposure n = 68400 (cut-off 09 Feb 2010), Pandemrix 0	No registration was done of pregnant women who were vaccinated. Currently a study is being undertaken to estimate the vaccine coverage and to determine if the vaccine coverage depended on gestational age or underlying illness. The study is being conducted by the RIVM and 14,000 women will be approached for this study.	

Norway (61430)	An estimated 70% of the pregnant women were vaccinated. Monthly for Celvapan (49) for WCBA and Pandemrix (377528) for WCBA.	1) 50 to 74%. The estimates is based on a small web-based request to general practitioners, http://www.uib.no/isf/eyr 2) 75 to 99%. Based on vaccination registers vs. registers of pregnant women. In the national registering system for vaccines there is no box for pregnancy. The Pregnancy record, in use by all pregnant women is being filled in manually.	

Poland (415681)	No vaccinations took place	No vaccinations took place	

Portugal (99896)		0 to 24%. Based on national registry. Available national data from ACSS - Administração Central do Sistema de Saúde, IP	

Slovakia (61158)	No exposure data available		

Slovenia (21746)	262 vaccinated total		

Spain (494944)	Weekly for Panenza (39183).	0 to 24%. Based on personal estimation.	4.7% uptake in Castilla and Leon regions [13].

Sweden (111076)	15195 pregnant women and 264326 WCBA were vaccinated in Stockholm county up to Feb 2010.		

Switzerland (77789)		Unknown to registry.	

United Kingdom (788417)	103680 doses of Pandemrix and 1141 doses of Celvapan. Likely an underestimation.	1) 50-74% were offered the vaccine according to primary care data from a UKTIS study [16]. 2) 25 to 49%. Guess/Estimate based on proportion of regional clinical at risk groups vaccinated.	Out of 2,061 women pregnant between September 2009 and January 2010, 64.8% were offered vaccination and 25.2% were vaccinated [16].

Note the questions asked differed slightly between the NUI and the EUROCAT questionnaire. The competent authorities were asked to provide any weekly or monthly vaccination data that were available. The EUROCAT member were asked to estimate the percentage of pregnant women vaccinated (see methods) and were requested to provide any vaccination data they had available.

WBCA: Women of childbearing age.

* Annual births 2009 given for comparison with numbers vaccinated. Note that numbers eligible for vaccination is the sum of the pregnant women in the relevant stage of pregnancy at initiation of the vaccination campaign plus the pregnant women entering the relevant stage of pregnancy during the vaccination campaign.

### Data on exposure of women of childbearing age to swine flu vaccine

Four countries were able to provide a figure on WCBA vaccinated (table 3).

## Discussion

The majority of countries (83% of 24 respondents) had an official vaccination policy for pregnant women. No replies were received from nine countries and it is possible that response bias results from smaller countries and countries without vaccination policies being less likely to respond. Twenty one countries out of the 27 in the EU, corresponding to 93% of the member population, were covered by the combined responses to both questionnaires.

Some countries had existing advance purchase agreements with pharmaceutical companies which allowed early procurement. A lack of capacity to identify and prioritise key population groups quickly may also have influenced policy decisions. Public perceptions, which can be influenced by certain key events in a population, may be significant. For example, in the Czech Republic, a previous key event involving vaccines contaminated with deadly live H5N1 avian flu virus, might have influenced the decision not to formulate an official vaccination policy [17].

A larger number of European countries have a vaccination policy for pregnant women against pandemic influenza than against seasonal influenza. Ten out of 27 (37%) European countries recommended vaccination against seasonal influenza for pregnant women during the 2008-9 influenza season: Austria, Belgium, Cyprus, Denmark, Estonia, Ireland, Italy, Portugal, Slovakia and Spain [18]. None of these countries could provide information on vaccine coverage of pregnant women. All these countries had a pandemic vaccination policy in force during the pandemic influenza, and it is possible that this was facilitated by the previous seasonal flu policy. Both in Canada and the United states, vaccination of pregnant women is recommended during the influenza season, while in Australia, vaccination of pregnant women in the second or third trimester of pregnancy is recommended during the influenza season [1].

Vaccination policy only partially determines what happens in a country. Data collected by the questionnaires demonstrated that countries with similar vaccination policies achieve very different vaccination rates. For example Italy and the Netherlands had similar vaccination policies but 23,016 women in the second and third trimester of pregnancy were vaccinated in Italy (population ~61 million) and 68,400 in the Netherlands (population ~17 million). The differences in vaccination rates detected between countries with similar vaccination policies might be related to different public perceptions of factors such as the vaccination campaign, vaccine safety and the risk of the influenza pandemic. In addition, inclusiveness and funding of different health systems and implementation issues associated with the delivery of different vaccination policies and associated education campaigns might have been influential. Furthermore, the decision to use vaccination centres instead of general practitioners for vaccination may have lowered the uptake of vaccination in some countries, such as France [19].

Varying pandemic influenza vaccine uptake rates have been reported for pregnant women. In our survey, three countries reported uptake above 50%. Uptake by pregnant women was reported as 6.9% in an Australian survey [20], 25.2% in a British survey [16], 76% in a Canadian survey [21], 22.7% and 37.1% in French surveys [9,12] and 8.8% in a German survey [15]. Factors associated with vaccine uptake in the Canadian survey were late stage of pregnancy, belief in the efficacy of the vaccine, and consultation of the (Canadian) pandemic influenza website. Factors negatively associated with vaccine uptake in the Canadian survey were belief that the vaccine has not been adequately tested and consultation of popular websites. The Australian survey concluded the main reasons for the low vaccine uptake were the vaccine frequently not being offered by the general practitioner and safety concerns both of the women and the general practitioners. In the British study, vaccination was offered to 64.8% of the pregnant women. In their review of the pandemic experience, the ECDC state the lack of vaccine acceptance can partially be attributed to the complex risk message communicated. This risk message communicated on the one hand that for people outside risk groups the risk of severe disease was low, but on the other hand it communicated that there was a small but real risk of severe disease and death for healthy adults and children [22]. In one American study, vaccination rates of pregnant women with seasonal influenza vaccine were shown to increase from 19% to 31% through increasing patient awareness via education of vaccination providers and posters, underlining the impact of patient awareness in vaccination campaigns [23]. A literature review found low vaccination rates of pregnant women in countries where seasonal influenza vaccination is recommended, ranging from > 0.1% to 12.8% [24].

While in most countries it was decided to vaccinate pregnant women of the second and third trimester, one of the main differences between vaccination policies was the decision whether to vaccinate pregnant women in the first trimester of pregnancy. This difference between countries on whether to vaccinate first trimester pregnant women has also been witnessed in seasonal influenza vaccination policies [1]. The first trimester of pregnancy is the period of major organogenesis and thus important in relation to risk of congenital anomalies, increasing the potential risk of vaccination. In addition, women in the first trimester of pregnancy are less susceptible to adverse health outcomes caused by influenza infection, decreasing the benefit of vaccinating this group. Differences in trimesters covered by the pandemic vaccination policy might have been caused by differences in appreciation of available safety and efficacy data, or more operational reasons such as potential for limited length of vaccine efficacy and wanting it to be most effective in the later two trimesters, or deciding that targeting women regardless of trimester would better ensure high coverage by the start of the second trimester. Post-pandemic immunogenicity data on Pandemrix in healthcare workers showed that three months post vaccination, 84% of the participants still had a protective antibody titre (HI titre ≥40) compared to 97% three weeks post vaccination [25]. Both figures are still well above the Committee for Medicinal Products for Human use (CHMP) criteria for seasonal vaccines of > 70% of the subjects achieving an HI titre of ≥40.

Whatever the policy regarding first trimester vaccination, where the entire population is offered vaccine, this will include WCBA who are in the early stages of pregnancy but do not yet know they are pregnant. Other target groups such as women with asthma, or health care workers and persons in close contact with vulnerable groups would also include women in very early pregnancy. These women need to be taken into account when monitoring vaccine safety in pregnancy. In addition, 1^st ^trimester pregnant women belonging to priority groups or specifically requesting for vaccination, could receive the vaccination in some countries that did not routinely vaccinate 1^st ^trimester pregnant women.

Differences between countries were found in vaccines used in pregnancy, including use of adjuvanted or non-adjuvanted vaccines and Thiomersal content. Some European pandemic vaccines were developed and registered pre-pandemic using a "mock-up" registration procedure [26]. This procedure assumes that swapping the strain in a vaccine will not substantially affect the safety and efficacy of the vaccine. The mock up vaccines would be authorized once a pandemic is declared, after which the initial strain is swapped for the pandemic influenza strain. Preliminary data on the immunogenicity of the monovalent non-adjuvanated and MF59-adjuvanated vaccines showed favorable results [27,28]. These clinical trials also demonstrated a single dose of the pandemic vaccine would be sufficient for an immunogenic response, allowing for antigen saving vaccination strategies. Several countries had established "advance purchase agreements" with manufacturers, leading to some of the differences in vaccine chosen [26]. In these contracts, the manufacturer agrees to supply its pandemic influenza vaccine as soon as possible after a pandemic has been declared and agree to reserve an agreed number of doses for the country. Country of origin of the manufacturer might have played a role in vaccine of choice, as is illustrated by Hungary making use of Fluval P, manufactured by the Hungarian company Omninvest. Other possible factors in choice of vaccine might originate in limited supplies available and differing appreciation of available safety and efficacy data. Several of the pandemic vaccine formulations included oil-in-water adjuvants (AS03 for Pandemrix and MF59 for Celtura and Focetria) or aluminium phosphate gel adjuvants (Fluval P) as an antigen saving policy [29] and to enhance the immunogenicity of the pandemic vaccine. Celvapan, CSL Pandemic Influenza Vaccine and Panenza did not contain adjuvants. Safety information of AS03 and MF59 adjuvants is sufficient in the general population, but limited in pregnancy [29,30]. Five countries solely used non-adjuvanted vaccine to vaccinate pregnant women. Some other countries used non-adjuvanted vaccines for the entire population (table 2). Celvapan was the only pandemic vaccine not to contain Thiomersal. No consensus has been reached on the risk of prenatal exposure to Thiomersal [31]. Both the AS03 and MF59 adjuvants contain squalene [29,32]. We included questions about the timeframe of the vaccination policy. There was variation in the speed of response and in at least one case the vaccines arrived after the vaccination campaign had been initiated. Vaccination campaigns were initiated between 28^th ^September 2009 and 27^th ^December 2009, while the first pandemic wave hit Europe in September-October 2009.

Post marketing surveillance of vaccines and medicinal products is essential, in particular with regard to their use in pregnancy since pregnant women are excluded from clinical trials and much of the prior safety testing. There are a number of different ways of carrying out post marketing surveillance, including case-control studies of adverse pregnancy outcomes, and cohort studies of women receiving the vaccine, but it is also essential to collect basic information about exposure of pregnant women in the population to the vaccine in order to interpret any possible changes in population rates of adverse pregnancy outcomes. The limited availability of exposure data and the variation in quality of the available exposure data collected on pregnant women in relation to the pandemic vaccine are striking, but in line with the observed lack of exposure data of pregnant women to seasonal influenza vaccination in Europe [18,33]. This problem is further complicated by the need to distinguish between first trimester of pregnancy and second and third trimester of pregnancy, when exploring the relationship between influenza vaccination and congenital anomalies. Pre-pandemic safety data of the pandemic vaccines and adjuvants used in the pandemic vaccines in pregnancy is fairly limited. In order to conduct population based pharmacovigilance, exposure data is needed to interpret congenital anomaly rates. A number of countries reported cohort studies being conducted in pregnant women e.g. a national cohort in Norway. The lack of comprehensive data on exposure of pregnant women to pandemic influenza vaccine also limits the policy evaluation process, which is required in order to learn from the vaccination efforts for future pandemic infectious disease outbreaks and vaccination campaigns.

## Conclusions

Overall, we conclude pandemic vaccination policies for pregnant women in Europe were heterogeneous and achieved varying vaccination rates of pregnant women. Only limited data on exposure of pregnant women to pandemic influenza vaccine was available in Europe. For the evaluation of vaccine safety and adjuvants safety in pregnancy and pandemic vaccination campaign effectiveness, increased monitoring of pandemic influenza vaccine coverage of pregnant women is required.

## Competing interests

The authors declare that they have no competing interests.

## Authors' contributions

Design of survey: JML and HD. Execution of survey and collation of responses: JML. Drafting the paper: JML, HD and GJM. This manuscript has been read and approved by all authors.

## Pre-publication history

The pre-publication history for this paper can be accessed here:

http://www.biomedcentral.com/1471-2458/11/819/prepub
